# Subcritical Fluid Chromatography at Sub-Ambient Temperatures for the Chiral Resolution of Ketamine Metabolites with Rapid-Onset Antidepressant Effects

**DOI:** 10.3390/molecules24101927

**Published:** 2019-05-19

**Authors:** Robert K. Hofstetter, Felix Potlitz, Lukas Schulig, Simon Kim, Mahmoud Hasan, Andreas Link

**Affiliations:** 1 Pharmaceutical and Medicinal Chemistry, Institute of Pharmacy, Friedrich-Ludwig-Jahn-Str. 17, 17489 Greifswald, Germany; felix.potlitz@gmail.com (F.P.); lukas.schulig@uni-greifswald.de (L.S.); 2 Department of Trauma, Reconstructive Surgery and Rehabilitation Medicine, University Medicine Greifswald, Ferdinand-Sauerbruch-Straße, 17475 Greifswald, Germany; kims@uni-greifswald.de; 3 Leibniz Institute for Plasma Science and Technology (INP Greifswald), Felix-Hausdorff-Straße 2, 17489 Greifswald, Germany; 4 Department of Clinical Pharmacology, Center of Drug Absorption and Transport (C_DAT), University Medicine Greifswald, 17475 Greifswald, Germany; hasanm@uni-greifswald.de

**Keywords:** supercritical CO_2_, sub-ambient temperature, subcritical fluid chromatography, chiral resolution, immobilized, coated, chiral stationary phase, ketamine, metabolites, depression

## Abstract

Chiral metabolites of ketamine exerting rapid-onset yet sustained antidepressant effects may be marketed directly in the future, but require chemo- and enantio-selective chromatographic methods for quality assurance and control. The chromatographic behavior of *S*-/*R*-ketamine, *S*-/*R*-norketamine, *S*-/*R*-dehydronorketamine, and (2*R*,6*R*)-/(2*S*,6*S*)-hydroxynorketamine in supercritical fluid chromatography (SFC) was investigated computationally and experimentally with the aim of identifying problematic pairs of enantiomers and parameters for chiral resolution. Retention on three different polysaccharide-based chiral stationary phases (Lux Amylose-2, i-Amylose-3, and i-Cellulose-5) provided new information on the significance of halogen atoms as halogen bond donors and hydrogen bond acceptors for enantioselectivity, which could be corroborated in silico by molecular docking studies. Modifiers inversely affected enantioselectivity and retention. Methanol yielded lower run times but superior chiral resolution compared to 2-propanol. Lower temperatures than those conventionally screened did not impair phase homogeneity but improved enantioresolution, at no cost to reproducibility. Thus, sub-ambient temperature subcritical fluid chromatography (SubFC), essentially low-temperature HPLC with subcritical CO_2_, was applied. The optimization of the SubFC method facilitated the chiral separation of ketamine and its metabolites, which was applied in combination with direct injection and online supercritical fluid extraction to determine the purity of pharmaceutical ketamine formulations for proof of concept.

## 1. Introduction

On 5 March 2019, ketamine (K) was approved by the FDA for treatment-resistant depression [1]. Unique in its mechanism of action and different from monoaminergic modulators, which alleviate depressive systems within 6–8 weeks and in only about 2/3 of patients, K exerts rapid onset (within hours) yet sustained (one week and more) antidepressant effects, even in patients suffering from treatment-refractory depression [2]. Placebo-controlled studies suggest that the sub-anesthetic infusion of *rac*-K lowers suicidal ideation in patients suffering from bipolar depression to a greater degree than midazolam (used to address functional unblinding due to dissociative effects associated with verum administration), and demonstrated memory improvement and pre- to post-infusion decrease in serum brain-derived neurotrophic factor (BDNF) as promising biomarkers [3].

However, *rac*-K infusion is known to induce anxiety, which appears to be associated with higher non-responder rates (up to 45%), defined as a less than 50% reduction on the Montgomery–Asberg Depression Rating Scale (MADRS) [4]. Euphoric and dissociative effects, on the other hand, increase abuse liability of K, and although actual prevalence is not known, the incidence of recreational use among young adults is estimated to be as high as 4.5%, which might include attempts at self-directed therapy [5].

The side effects and abuse liability associated with the parent drug have motivated the pursuit of K-like alternatives such as enantio-pure, sustained-release or active metabolite formulations in order to circumvent the current limitations placed on antidepressant therapy with *S*-K, which is available only for supervised administration at certified health care providers, and *rac*-K, the off-label infusion of which is restricted to in-patients [6,7,8]. Regrettably, the nature and precise mechanism of action responsible for rapid-onset yet sustained antidepressant effects is still being debated [9].

Both *R*- and *S*-K exert anesthetic effects by the non-competitive antagonism of glutamatergic *N*-methyl-D-aspartate receptors (NMDARs), with the *S*-enantiomer being approximately 3–4-fold more potent than the *R*-enantiomer. While the NMDAR hypothesis therefore predicts greater efficacy of *S*-K as well as similar effects for non-K NMDAR subtype inhibitors, antidepressant-predictive animal models have indicated *R*-K to be the more potent antidepressant [10].

The identification of metabolites with distinctive pharmacodynamics has provided alternative hypotheses to K′s mechanism of antidepressant action: demethylation yields *R*- and *S*-norketamine (*R*-/*S*-NK), which may undergo oxidation to yield unsaturated *R*- and *S*-dehydronorketamine (*R*-/*S*-DHNK) and hydroxylated (2*R*,6*R*)- and (2*S*,6*S*)-hydroxynorketamine (*RR*-/*SS*-HNK). The local blockade of NMDAR at the anti-reward center by K [11] or *S*-NK [12] may account for rapid-onset antidepressant effects, whereas synaptogenesis or BDNF modulation could exert long-term antidepressant effects [13]. *RR*-HNK, on the other hand, was shown to induce antidepressant effects through early and sustained activation of α-amino-3-hydroxy-5-methyl-4-isoxazole propionic acid receptors (AMPARs) at levels insufficient for NMDAR inhibition [14], but may converge on the same downstream pathways, including BDNF modulation [15].

Interestingly, pharmaceutical formulations of these metabolites alleviated the detrimental effects associated with K administration in animal studies, as the parent drug *rac*-K is largely responsible for dissociative side effects and abuse potential [2].

The necessity of enantio- and chemo-selective determination methods for the pharmaceutical quality control of individual metabolite formulations is clear from the striking differences and eudysmic ratios between K, NK, DHNK, and HNK.

Although HPLC has been the workhorse of industrial-scale quality control, supercritical fluid chromatography (SFC) has begun to encroach on quality assurance territory as a sustainable [16] and notably more cost-effective alternative to conventional chromatography [17]. The use of supercritical CO_2_ (scCO_2_) as a hexane-like mobile phase is accompanied by beneficial physical attributes (high mass transfer, high diffusivity, low viscosity), which favors the rapid separation of complex mixtures even at conditions below the critical point (subcritical or enhanced fluidity) [18], and therefore has been used for the separation of metabolites of K from urine [19].

The aim of this study was to identify and optimize the parameters responsible for the enantioseparation of K, as this racemate proved the most challenging to resolve on polysaccharide-based chiral stationary phases (CSPs). The choice of column and modifier were identified as major determinants while additives, pressure, and flow rate modulation had only minor effects on chiral resolution. Temperatures below the range conventionally screened provided a modest but essential improvement in selectivity over traditional SFC, which enabled the quality control of antidepressant *rac*-K and metabolite formulations.

## 2. Results

### 2.1. Stationary Phase Screening

**Figure 1:**
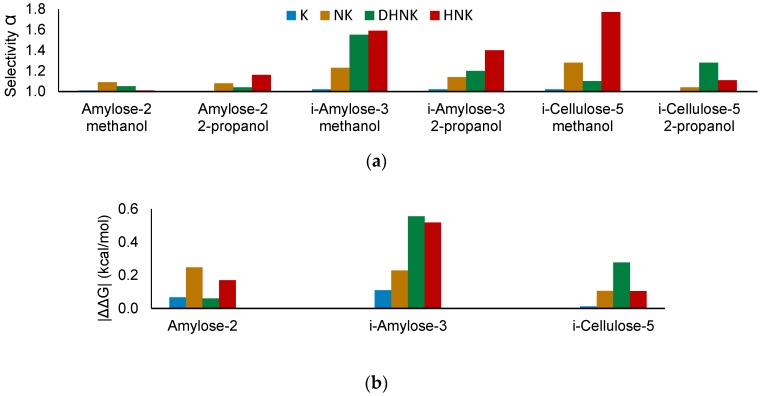
(**a**) Experimental screening for selectivity (α) on three chlorinated polysaccharide stationary phases for ketamine (K), norketamine (NK), dehydronorketamine (DHNK), and hydroxynorketamine (HNK), using supercritical CO_2_ (scCO_2_) modified by methanol or 2-propanol. (**b**) Calculated |ΔΔGG|-values (described in Section 2.2) enable the prediction of enantioseparation and aid the explanation of experimental results.

We previously reported on the challenges associated with the HPLC bioanalysis of ketamine metabolites, which entailed one achiral C18-phase (XTerra MS^®^) and two CSPs that were based on protein (Chiral-AGP^®^ for K, NK and DHNK) and polysaccharides (Lux^®^ Amylose-2 for HNK) [20]. When operated in SFC mode, the same polysaccharide-based CSP afforded more versatility, as it enantioselectively resolved all metabolites but the parent drug (K), which could not be fully separated even after optimization [19]. The development of immobilized CSPs in which the chiral selector is chemically bonded to the SiO_2_-particles has expanded physico-chemical compatibility [21] and in some cases provided additional selectivity and robustness for chiral separations [22]. In order to improve the resolution of *rac*-K, three chlorinated CSPs that differed in chemical modification and polysaccharide backbone were compared. Employing the parameters shown in supplementary information Figure S1, Lux Amylose-2 [coated tris(3-chloro-5-methylphenylcarbamate)amylose], i-Amylose-3 [immobilized tris(3-chloro-5-methylphenylcarbamate)amylose] and i-Cellulose-5 [tris(3,5-dichlorophenylcarbamate)cellulose] were included in the initial column scouting (Figure 1a).

The screening verified earlier observations of higher enantioselectivity (α) for the separation of demethylated metabolites NK, DHNK and HNK compared to the parent drug K. In comparison to coated amylose-2, however, immobilized i-Amylose-3 and i-Cellulose-5 exhibited higher chiral recognition, particularly when methanol was used. The highest α values were obtained for the hydroxylation product HNK, which was also separated on Amylose-2 but required the modifier 2-propanol. Albeit providing higher elution strength, the use of methanol as a modifier provided higher α on immobilized CSPs for most analytes, possibly due to conformational stabilization, and was therefore used in combination with i-Amylose-3 during further method development.

### 2.2. Molecular Modelling Studies

Computational methods were utilized to investigate differences in the three-dimensional structure of the column polymers and possible interactions of the chiral recognition mechanism at the atomistic level by molecular docking.

In contrast to ligand–receptor interaction, there is no specific binding site for ligands on the polymer surface and the fluctuation between bound and unbound states is much larger. It is therefore hardly possible to calculate accurate quantitative energy contribution using molecular docking, even if solvent effects are taken into account. Nevertheless, it is a valuable tool to predict qualitative assertions and possible interaction with the polymer, as previously published in literature [23]. To gain a deeper understanding of the dynamic processes and the solvent effects, large scale molecular dynamics simulations will be a part of future work.

**Figure 2:**
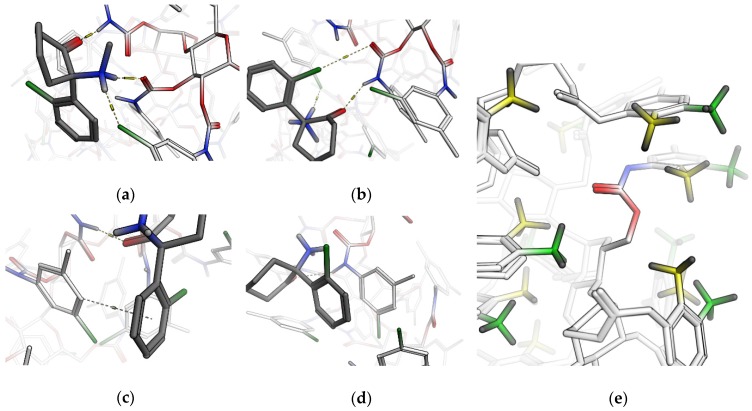
Representative poses of *R*/*S*-ketamine to depict possible interaction types: (**a**) Hydrogen bonding including halogen acceptors, (**b**) halogen bonds, (**c**) CH–π bonds and (**d**) π–π interaction. (**e**) Superposition of Amylose-2 and i-Amylose-3 with methyl groups colored in yellow and green, respectively. The substituents in position 2 of the benzene ring sterically restrict access to the carbamate for hydrogen bonding.

We prepared the three-dimensional structure of the three column types as hexamers and both enantiomers for all ligands according to the description in the Methods section. By using a blind docking approach, 25 docking poses were obtained and sorted by binding free energy to create a representative set of possible interaction sites. The main types of interactions are hydrogen bonds (donor/acceptor, including halogen acceptors), π–π, halogen, and CH–π bonds (Figure 2a–d).

In Table 1, the energy differences for the highest docking score between the two enantiomers |ΔΔG| were calculated in order to predict chiral recognition on all three modified polysaccharide-based CSPs. As illustrated in Figure 1b, higher |ΔΔG| values are in good agreement with the experimentally determined enantioselectivity α.

Since the only substitutional difference between Amylose-2 and i-Amylose-3 is the position of the methyl group attached to a phenyl carbamate, we flexibly aligned both oligomers to get a better understanding of how this affects the three-dimensional structure. As shown in Figure 2e, the methyl groups of Amylose-2 (colored in yellow) sterically restrict accessibility to the carbamate (colored by atom), and thus prevent possible hydrogen or halogen bonding. To further investigate these interactions relating to chiral recognition, *RR*-HNK and *SS*-HNK were docked into the same site on i-Amylose-3, as it was known from experimental data which enantiomer had the higher residence time and HNK also had the highest enantioselectivity value on this phase.

**Figure 3:**
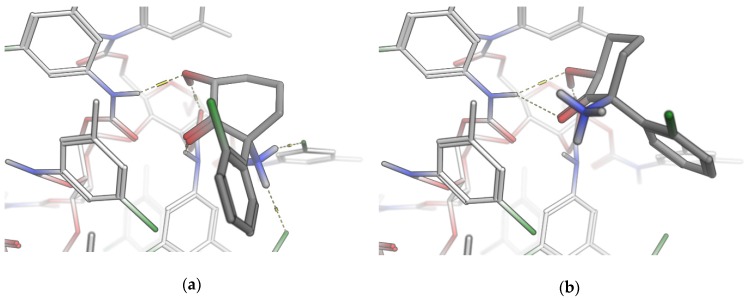
Docking poses of *RR*-HNK (**a**) and *SS*-HNK (**b**) at a defined binding site. The three-dimensional geometry of the *RR*-enantiomer permits a larger hydrogen bonding network, stronger hydrophobic interactions and therefore extended residence time. (|ΔΔG| = 2.1 kcal/mol).

The final docking poses are shown in Figure 3. The *RR*-enantiomer is buried deeper in the oligomer surface while maintaining strong hydrogen bonding between the carbonyl and hydroxyl groups. Two additional hydrogen bonds are possible between the primary amino group and the chlorine substituents acting as acceptor atoms. As opposed to the *SS*-enantiomer, all hydrogen bond donors of the RR-HNK are saturated and not available for interactions with carbon dioxide (Figure 3). Chiral recognition appears to be based on the ability of one enantiomer to form stronger hydrogen bond networks with the stationary phase than its counterpart.

### 2.3. Effects of Elution Mode

The miscibility of scCO_2_ with polar co-solvents (modifiers) can be exploited to adjust mobile phase solvation strength. The critical point of such mixtures is generally beyond the pressure and temperature capacities of commercially available platforms, while the favorable fluid characteristics are retained and thus the term SFC is used for both super- (pure scCO_2_) and subcritical (modified scCO_2_) mobile phase compositions containing more CO_2_ than modifier (as expressed by volumetric control) [24]. Because this terminology has its obvious flaws, Otsubo et al. avoid the term SFC for low-temperature HPLC using pure liquid carbon dioxide as the mobile phase in subcritical state [25]. The expansion of modifier use has led to the development of ‘enhanced-fluidity liquid chromatography′ (EFLC), where mobile phase proportions are switched (modifier > scCO_2_) to allow for the separation of analytes as polar as proteins, nucleosides, and sugars [26,27,28]. Since elution patterns may vary between SFC, non-polar HPLC with pure CO_2_, EFLC [29], and polar organic HPLC (100% polar eluent) [30], isocratic elution with different CO_2_/methanol ratios was used to study the effects of elution modes on ketamine metabolites and to identify the most promising mode for enantioseparation (Table 2).

**Figure 4:**
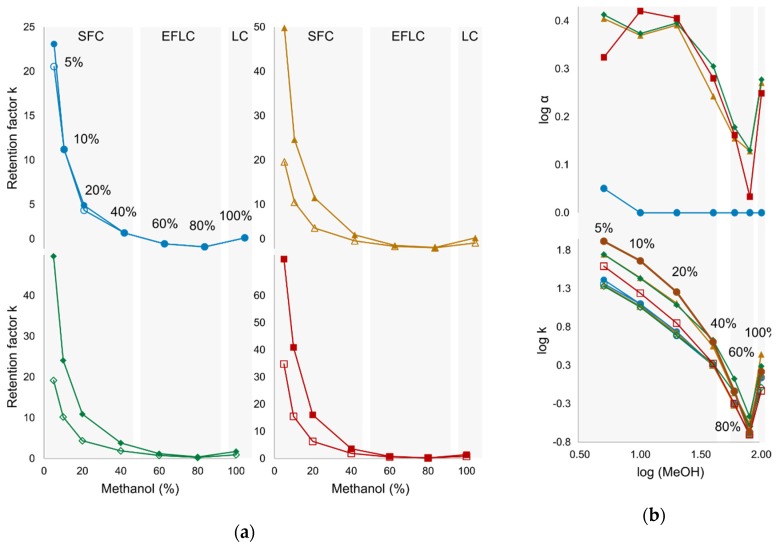
(**a**) Change in retention k for racemates of ketamine (blue), norketamine (orange), dehydronorketamine (green), and hydroxynorketamine (red) during transition from supercritical fluid chromatography (SFC) (5–40% methanol) to enhanced-fluidity liquid chromatography (EFLC) (40–80%), and ultimately HPLC mode (100%). (**b**) The trend of decreasing enantioseparation log α and log k with rising modifier concentration continues to 80%, at which point retention and separation increase and elution order is reversed. Column: Lux i-Amylose-3; modifier: methanol; additive: none.

In the presence of scCO_2_ (SFC, EFLC), rising modifier percentages increased the elution strength, which led to a decrease in retention for all analytes. Transition to HPLC, however, was associated with a trend reversal (higher retention/resolution at 100% compared to 60% methanol), leading to an apparent retention minimum within EFLC at 80% (Figure 4). Interestingly, the transition from SFC to EFLC was accompanied by a reversal of elution order: in SFC mode, K and NK eluted first and HNK last (K ≈ NK > DHNK > HNK); in EFLC mode, the retention of HNK began to fall below that of NK and DHNK, most likely due to the increase in hydrogen bond availability in the mobile phase (hydroxyl group of methanol) as this type of interaction was found to dominate retention and chiral recognition of HNK on Lux i-Amylose-3 (Figure 2a and Figure 3). Since K was deemed the most challenging target racemate, further efforts focused on the SFC mode at low modifier gradients, as the isocratic screening yielded the highest α value for K at 5% methanol.

### 2.4. Mobile Phase Optimization

**Figure 5:**
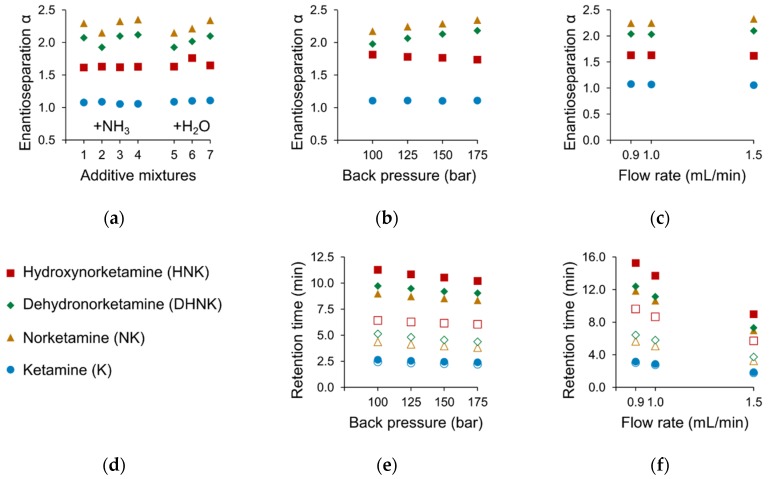
Effect of additives (**a**), back pressure (**b**), and flow rate (**c**) on the enantioseparation of ketamine metabolites (**d**), and the effects of back pressure (**e**) and flow rate (**f**) on retention times. Column: Lux i-Amylose-3; modifier: methanol; additive: NH_3_, 0.015% (1), 0.0375% (2), 0.075% (3), 0.15% (4); H_2_O, 0% (5), 1% (6), 5% (7).

The use of 0%, 0.015%, 0.0375% or 0.15% of the basic additive NH_3_ (aqueous solution) reduced retention while improving enantioseparation α up to a concentration of 0.0375%. Additional water content (0%, 1%, 5%, 10%) neither improved nor impaired separation but helped to reduce retention times (Figure 5a). The exploitation of this effect was limited however by on-column pressure buildup which restricted the usage of 10% H_2_O to low flow rates.

A back pressure increase from 100 to 175 bar benefited the α values for NK and DHNK, but exerted only minor effects on K and HNK. Similarly, the variation of flow rate (tested from 0.5 to 1.5 mL/min) shortened run times but had only a small impact on separation. The maximum pressure tolerance of the stationary phase (310 bar) limited the use of higher flow rates. Back pressure was therefore set to the minimal value of 100 bar in order to enable flow rates of 1.5 mL/min in the final method. As illustrated in Figure 5, the optimization of additives, pressure, and flow rate exerted only minor effects and did not facilitate the desired result of separating *rac*-K.

### 2.5. Temperature Effects

**Figure 6:**
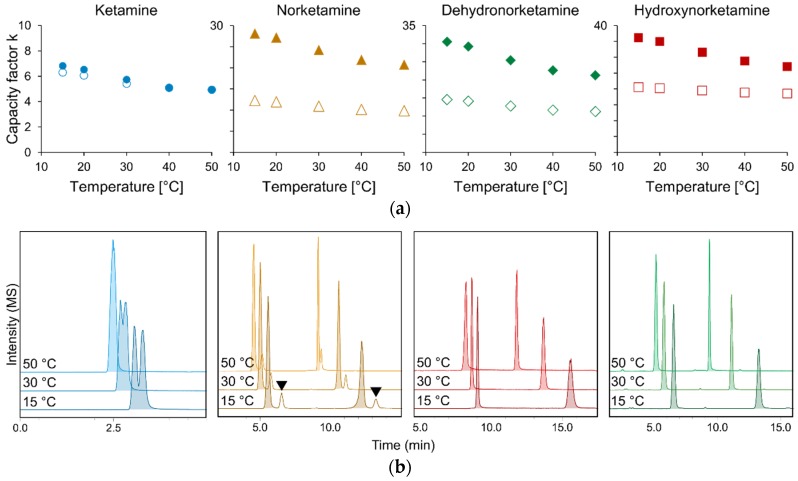
Effects of temperature variation on capacity factors k (**a**). Representative MS-chromatograms illustrate the increase in enantioresolution for all analytes at lower temperatures (**b**). Note the isobaric interference of ^37^Cl-dehydronorketamine (black marks) in the mass trace of ^35^Cl-norketamine (orange), the chemoselective separation of which is also achieved by sub-ambient temperature subcritical fluid chromatography (SubFC). Column: Lux i-Amylose-3; modifier: methanol; additive: NH_3_ (0.0375%).

The effect of temperature was investigated between 15 and 50 °C. As predicted by Pirkle [31], lowering the column temperature to sub-ambient temperatures increased retention for all analytes and improved chiral separation by predominantly affecting the second eluting enantiomer (Figure 6). With the exception of the choice of stationary phase and modifier, the effects of temperature were more pronounced than those observed by the variation of additive, pressure, or flow rate.

### 2.6. Mobile Phase Homogeneity

**Figure 7:**
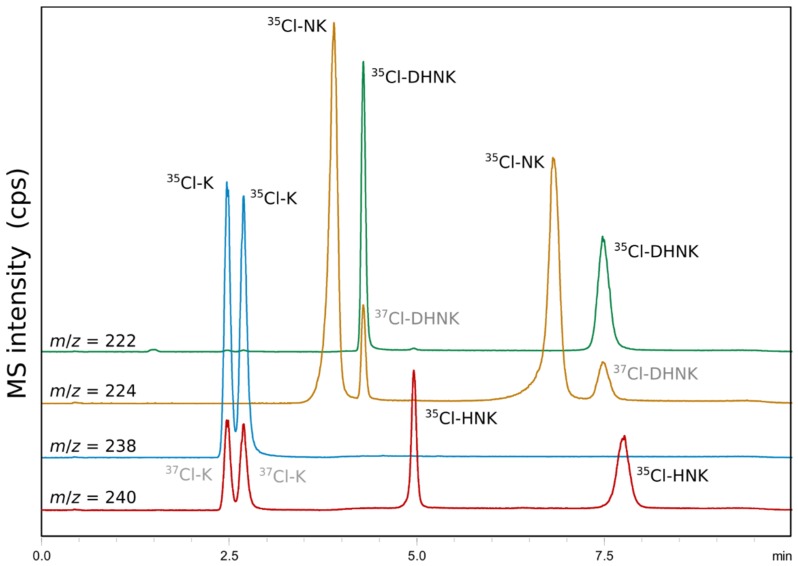
Representative chromatogram obtained after the optimization of the sub-ambient temperature subcritical fluid chromatography (SubFC) method for chemo- and enantio-selective separation of ketamine (K), norketamine (NK), dehydronorketamine (DHNK), and hydroxynorketamine (HNK). Note the isobaric interference of ^37^Cl-isotopic analytes between mass traces that differ in *m*/*z* 2, thus requiring chemo-selective separation even when using MS detection. Column: Lux i-Amylose-3; modifier: methanol; additives: NH_3_ (0.0375%), H_2_O (5%); back pressure: 100 bar; temperature: 15 °C.

The supercritical point of pure scCO_2_ is situated at 31 °C and 74 bar, but the introduction of modifier and additives raises these parameters so that the term SFC paradoxically includes subcritical fluid chromatography [32]. Pirkle suggested the term SubFC for subcritical fluid chromatography to avoid confusion, but because sometimes SFC turns into SubFC unnoticed within a gradient elution, SFC and SubFC are most often not distinguished. SubFC, principally HPLC with CO_2_, retains many of the beneficial separation attributes of SFC as long as phase homogeneity is maintained to yield reproducible retention times. A 10-run reproducibility test was performed on all analytes at 15 °C in order to screen for variations in retention times due to phase separation. As shown in Table 3, the relative standard deviation (RSD) of retention times varied between 0.4% (HNK) and 1.4% (K), and thus was similar to alternative forms of SFC [28]. A representative chromatogram obtained by the final method can be seen in Figure 7.

### 2.7. Application to Antidepressant Drug Formulations

**Figure 8:**
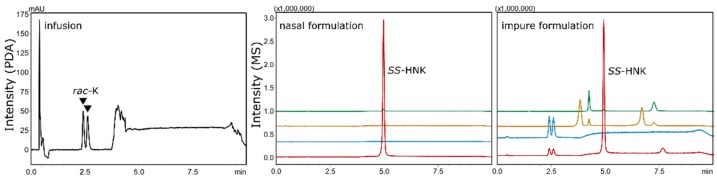
Quality control of a *rac*-K infusion using a PDA detector (SubFC–UV at 200 nm) and a nasal spray formulation of pure and adulterated (2*S*,6*S*)-hydroxynorketamine (*SS*-HNK) in combination with mass detection (SubFC–MS in SIM mode).

The direct injection of a *rac*-K infusion enabled the determination of the racemic nature of the antidepressant within as little as 3 min by sub-ambient temperature subcritical fluid chromatography (SubFC) and photodiode array (PDA) detection. Due to the stronger retention of demethylated metabolites, a gradient starting at 3.25 min was used in the final method. The change in mobile phase UV-absorption interfered with the PDA-detection of NK, DHNK and HNK, which required MS-detection. The enantiopurity of an *S*-HNK nasal spray formulated for research purposes was therefore analyzed by SubFC–MS. Pure (single peak at 5 min for HNK, *m*/*z* 240) and contaminated formulations (2%, in analogy to K [33]) could be easily discriminated (Figure 8).

**Figure 9:**
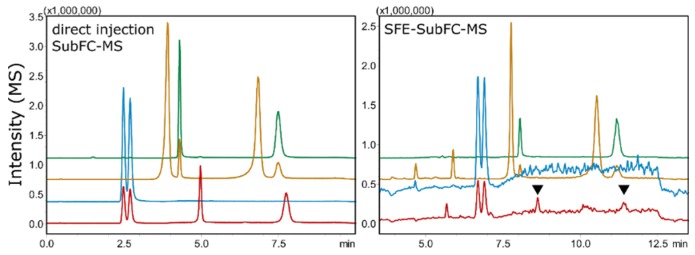
Comparison of direct injection SubFC–MS and online scCO_2_-extraction–chromatography SFE–SubFC–MS of ketamine (blue), norketamine (orange), dehydronorketamine (green), and hydroxynorketamine (red). While the former analytes are easily extracted, hydroxynorketamine (black marks) is more polar and is only incompletely extracted using pure scCO_2_.

For formulations containing ingredients that were not deemed suitable for direct injection, supercritical fluid extraction (SFE) hyphenated to SubFC–MS was performed on adsorbed samples. K-, NK-, and DHNK-formulations were found to be easily extractable using pure scCO_2_, whereas the more polar HNK is known to require the addition of polar additives for complete extraction (Figure 9) [34].

## 3. Discussion

Although SFC separations on the latter CSP have been reported [35,36,37], to the best of our knowledge this is the first comparison involving i-Amylose-3. Our results suggest halogen- and hydrogen-bonding to be significant for chiral recognition of chlorinated analytes such as K and its metabolites on chlorinated polysaccharide-based CSPs, which is supported by previous observations concerning achiral chemo-selectivity of halogenated analytes on Amylose-2 [38].

As became clear during method development, the use of additives and the variation of pressure and flow rate showed only minor effects on resolution. Changes in temperature, however, significantly impacted chiral separation. Contrary to previous reports of peaks narrowing as temperature rises [22], we observed better peak shapes and chiral resolution as temperature declined with the best performance at sub-ambient temperatures (15 °C). Method development for SFC methods generally entails the screening of temperatures from 25–60 °C [39] and sometimes ambient temperatures [37,40], however, sub-ambient temperature working conditions, here tentatively termed SubFC, proved superior in the case of analytes that otherwise resisted chiral resolution (K). Indeed, while higher temperatures are known to affect the chiral recognition ability of polymeric CSPs through the induction of—often irreversible—conformational changes [41], lower temperatures may increase the rigidity of the polysaccharide back bone resulting in higher accessibility to the chiral ravines, within which enantio-selective hydrogen bonding is responsible for chiral recognition [42]. Our findings support the significance of hydrogen bonding in SFC mode at lower temperatures, and indicate higher performing yet reproducible results that will require further verification with a broader range of analytes and stationary phases.

The final separation method was chemo-selective to K, NK, DHNK, and HNK. The method exhibited enantio-selectivity for all metabolites. The separation of *rac*-K remained the most challenging enantiomer pair and although baseline separation was not achieved, resolution was higher than that of previous SFC methods, which allowed the evaluation of enantiomeric excess [19].

The application of SubFC–UV and SubFC–MS to pharmaceutical products demonstrated compatibility with the direct injection of aqueous formulations and online supercritical fluid extraction–supercritical fluid chromatography of adsorbed samples (which in our lab is used for testing for contamination/residues). As SFE has recently gained importance as a swift and safe offline extraction method for antioxidant [43], anti-metastatic [44], and bioactive pharmaceutical compounds [45], compatibility with online SFE may be considered indicative of SubFCs’ potential for quality control.

## 4. Materials and Methods

### 4.1. Chemicals

CO_2_ (99.995% purity) was provided by Air Liquide (Duesseldorf, Germany). Modifiers and additives were obtained in LC-MS grade from VWR (Leuven, Belgium). Lux Amylose-2 (150 × 4.6 mm, 5 µm), i-Amylose-3 (150 × 2.0 mm, 3 µm), and i-Cellulose-5 (100 × 3.0 mm, 3 µm) were purchased from Phenomenex (Aschaffenburg, Germany). *S*-/*R*-ketamine (*S*-/*R*-K), *S*-/*R*-norketamine (*S*-/*R*-NK), and *S*-/*R*-dehydronorketamine (*S*-/*R*-DHNK) were acquired as hydrochlorides from Sigma-Aldrich (Steinheim, Germany), except for (2*R*,6*R*)- and (2*S*,6*S*)-hydroxynorketamine (*SS*-/*RR*-HNK) which were kindly provided by the National Center for Advancing Translational Sciences (Rockville, Maryland, USA). Stock solutions were prepared in methanol and stored at −20 °C. Working solutions were prepared weekly and stored at 4 °C.

### 4.2. Instruments

**Figure 10:**
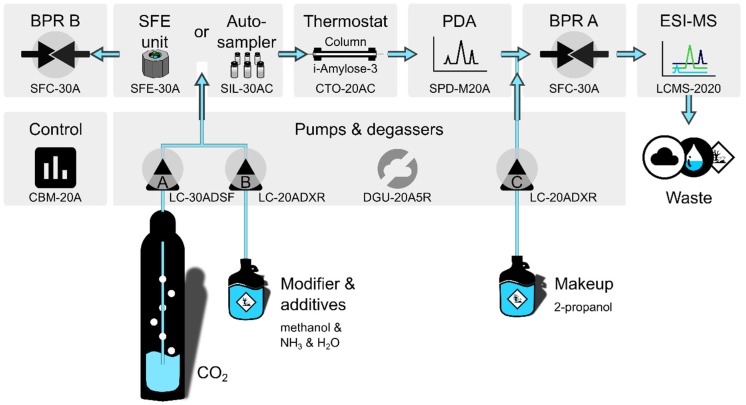
Schematic setup of the super-/subcritical fluid chromatograph. SFE, supercritical fluid extraction; PDA, photo diode array detector; BPR, back pressure regulator; ESI-MS, electrospray ionization–mass spectrometry.

Data acquisition was realized using a Nexera SFE-SFC/UHPLC switching system (Shimadzu Corporation, Kyoto, Japan) shown in Figure 10. The pumping system consisted of three units (one LC-30ADSF for liquid CO_2_ (A) and two LC-20ADXR for modifier (B) and make-up (C) delivery). Samples were introduced either directly (autosampler SIL-30AC) or by online supercritical fluid extraction (SFE-30A auto extractor equipped with 0.2 mL extraction vessels). The system consisted further of a column thermostat (CTO-20AC), a degasser (DGU-20A5R), a communications module (CBM-20A), and two back pressure regulators BPR A and B (SFC-30A). Only BPR A was used for the dynamic regulation of back pressure; the splitting function of BPR B (on-column/waste split for the analysis of highly concentrated biomatrices) was not used. The PDA detector (SPD-M20A) was set to 200 nm. Optimization of electrospray ionization–single quadrupole mass spectrometry (LCMS-2020) yielded 0.1 mL/min make up (2-propanol), 1.5 L/min nebulizing and 12 L/min drying gas (N_2_); 250 °C desolvation line, 300 °C heat block, and 350 °C interface temperature; 4.5 kV interface voltage. SIM: *m*/*z* 238 (*S*-/*R*-K), 224 (*S*-/*R*-NK), 222 (*S*-/*R*-DHNK), and 240 (*SS*-/*RR*-HNK). The system was controlled by Shimadzu LabSolution software (Version 5.91, Kyoto, Japan).

### 4.3. Chromatographic Parameters

Unless stated otherwise, the parameters of the final method apply. Stationary phase: i-Amylose-3 (150 × 2.0 mm, 3 µm). Mobile phase (A): supercritical CO_2_; modifier (B): methanol; additives (as a volumetric percentage of B): NH_3_ (0.0375%) and H_2_O (5%). Gradient (B): 0–3.0 min (8%), 3.0–3.25 min (8–25%), 3.25–8.5 min (25%), 8.5–9.0 min (25–8%), 9.0–10.0 min (8%). Flow rate: 1.5 mL/min. BPR: 100 bar. Temperature: 15 °C.

Stationary phases were screened using methanol or 2-propanol without an additive in gradient mode (Section 4.3). Flow rate was adjusted to reflect differences in column inner diameter (for details, see supplementary information Figure S1).

Since mobile phase viscosity increased with rising B%, total flow rates had to be adjusted during transition from SFC (5%, 2.5 mL/min; 10%, 2.0 mL/min; 20%, 1.5 mL/min; 40%, 1 mL/min) to EFLC (60%, 0.6 mL/min; 80%, 0.5 mL/min) and ultimately HPLC (100%, 2.5 mL/min). No additive was used in this experiment in order to prevent the damage of the stationary phase in HPLC mode, which—even when using immobilized materials—is less resistant to basic than to acidic pH.

Additive studies were performed in methanol due to the superior resolution in this modifier. NH_3_ (25% aqueous solution) was tested at the following concentrations: 0.015%, 0.0375%, 0.075%, and 0.150%. Values are given as percent volume ratio, e.g., in order to obtain a modifier solution containing 0.015% NH_3_, 30 µL of aqueous NH_3_ was added to 50 mL of methanol, stirred, and sonicated for 5 min. Effects of additional water were investigated by adding 1%, 5%, or 10% of water to a solution of methanol containing 0.0375% NH_3_.

### 4.4. Molecular Modelling

All calculations were performed using the Molecular Operating Environment (MOE) software suite (version 2019.01) [46]. For each column material, a hexameric strand was built of customized D-glucopyranose monomers by replacing the hydroxyl groups with the corresponding substituted phenyl carbamates in positions 2, 3 and 6. AMBER force field parameters were applied and the geometry was optimized using LowModeMD [47] to remove initial strains.

The 2D structures were prepared from SMILES, converted to 3D, energetically minimized and protonated according to a pH value of 5 [48]. AM1-BCC charges and AMBER force field parameters were applied prior to molecular docking.

Since no explicit binding site can be defined, a blind docking approach was utilized, where the whole hexamer is used as the receptor structure. Docking was performed in two steps. We selected 100 poses using flexible ligands, the Triangle Matcher placement method, and London dG scoring. A total of 25 poses for each ligand were refined, while keeping the oligomers rigid. All final poses were visually inspected afterwards.

Retention factors

k = (t_R_ − t_0_)/t_0_ (1)

were based on void times t_0_ estimated from the earliest baseline perturbation as described by Zhu et al. [49]. Resolution

R_s_ = (1.18 × (t_R1_ − t_R2_))/(w_h1_ + w_h2_) (2)

was calculated according to the European Pharmacopoeia from the difference of retention and the sum of peak width at half-height (w_h_) [50].

### 4.5. Application to Pharmaceutical Formulations

Infusions of *rac*-K were reproduced according to clinical use (0.5 mg/mL in isotonic NaCl-solution) and an experimental nasal spray formulation for non-human application containing *S*-HNK (1 mg/mL) was diluted 1:1000 prior to direct injection (supplementary information Figure S2). A master mix containing racemates of K, NK, DHNK, and HNK was prepared identically and either injected directly (for comparison) or adsorbed onto calcined SiO_2_/quartz (isolute^®^ HM-N) and dried for 1 h at 40 °C. Supercritical fluid extraction was performed statically for 3.0 min and dynamically for 0.5 min with pure scCO_2_. The extract was directly introduced into the chromatographic system but was trapped at the column head, as elution did not proceed in the absence of a modifier. SubFC was performed as described above. Details of the extraction program are given in the supplementary information (Figure S3).

## Figures and Tables

**Table 1:** Calculated |ΔΔG| and observed enantioseparation α using methanol ^1^ or 2-propanol ^2^ as a modifier.

Analyte	Amylose-2			i-Amylose-3			i-Cellulose-5		
	|ΔΔG| (kcal/mol)	α ^1^	α ^2^	|ΔΔG| (kcal/mol)	α ^1^	α ^2^	|ΔΔG| (kcal/mol)	α ^1^	α ^2^
K	0.07	1.01	1.00	0.11	1.02	1.02	0.01	1.02	1.00
NK	0.25	1.09	1.08	0.23	1.23	1.14	0.10	1.28	1.04
DHNK	0.06	1.05	1.04	0.56	1.55	1.20	0.28	1.10	1.28
HNK	0.17	1.01	1.16	0.52	1.59	1.40	0.10	1.77	1.11

**Table 2:** Retention times (Rt) and factors (k) of the first eluted enantiomer and enantioseparation α of the racemates for ketamine (K), norketamine (NK), dehydronorketamine (DHNK) and hydroxynorketamine (HNK) at different ratios of co-solvent B (methanol) and flow rates (FR) on Lux i-Amylose-3.

Mode	B	FR	t_0_	K			NK			DHNK			HNK		
	(%)	(mL/min)	(min)	Rt ^1^	k	α	Rt ^1^	k	α	Rt ^1^	k	α	Rt ^1^	k	α
SFC	5	2.5	0.23	4.98	23.1	1.1	4.76	49.8	2.5	4.66	49.7	2.6	8.27	73.4	2.1
	10	2.0	0.28	3.38	11.2	1.0	3.19	24.6	2.3	3.10	24.1	2.4	4.57	40.9	2.6
	20	1.5	0.37	2.10	4.6	1.0	2.12	11.5	2.5	2.01	10.9	2.5	2.73	16.1	2.5
	40	1.0	0.61	1.71	1.8	1.0	1.72	3.2	1.8	1.77	3.8	2.0	1.77	3.6	1.9
EFLC	60	0.6	0.90	1.40	0.6	1.0	1.39	0.8	1.4	1.61	1.2	1.5	1.41	0.8	1.5
	80	0.5	1.13	1.38	0.2	1.0	1.39	0.3	1.3	1.45	0.4	1.3	1.38	0.2	1.1
HPLC	100	0.4	1.32	2.96	1.2	1.0	3.08	2.5	1.9	2.53	1.7	1.9	2.42	1.5	1.8

^1^ Retention times of the first eluted enantiomer (min).

**Table 3:** Variation of retention times Rt for racemic ketamine (K), norketamine (NK), dehydronorketamine (DHNK), and hydroxynorketamine (HNK) during consecutive separations (*n* = 10) with sub-ambient temperature subcritical fluid chromatography (SubFC). SD, standard deviation; RSD, relative standard deviation.

Analyte	First Eluting Enantiomer		Second Eluting Enantiomer		Resolution
	Rt ± SD (min)	RSD (%)	Rt ± SD (min)	RSD (%)	Rs
K	2.466 ± 0.028	1.13	2.680 ± 0.038	1.44	1.33
NK	3.893 ± 0.017	0.45	6.793 ± 0.072	1.06	12.40
DHNK	4.281 ± 0.022	0.52	7.425 ± 0.090	1.25	16.42
HNK	4.956 ± 0.021	0.43	7.729 ± 0.042	0.54	13.04

## Supplementary Materials

The following are available online. Figure S1: System settings (column screening); Figure S2: System settings (SubFC); Figure S3: System settings (SFE- SubFC).
