# Supplementary material for: Subcritical Fluid Chromatography at Sub-Ambient Temperatures for the Chiral Resolution of Ketamine Metabolites with Rapid-Onset Antidepressant Effects
Source: Molecules. 2019 May 19;24(10):1927. doi: 10.3390/molecules24101927 (PMC6572699; doi:10.3390/molecules24101927)
Supplement: Supplementary file 1 [file molecules-24-01927-s001.zip › S1.pdf]

Sample Information

Data File : Amy-2_IPA.lcd

Method

<<Pump>>
Mode : Binary gradient
Total Flow : 3.0000 mL/min
Pump C Flow : 0.1000 mL/min
B Conc. : 8.0 %
B Curve : 0
PressMax : 350 bar
PressMin : 0 bar
Pump C PressMax : 350 bar
Pump C PressMin : 0 bar
Solenoid Valve B Name : LPGE Unit
Solenoid Valve B : A
Compressibility Setting : Off
Pump A Shut Off Valve : Open

<<Autosampler>>
Autosampler Model : SIL-30AC
Enable Autosampler : Use
Rinse Type : External only
Sample Rack : Rack 1.5 mL 105 vials
Rinsing Volume : 500 uL
Needle Stroke : 52 mm
Control Vial Needle Stroke : 52 mm
Rinsing Speed : 35 uL/sec
Sampling Speed : 5.0 uL/sec
Sample Discharge Speed : 1.0 uL/sec
Measuring Line Purge Time : 10.0 min
Rinse Port R0 Purge Time : 10.0 min
Rinse Mode : Before and after aspiration
Rinse Dip Time : 0 sec
Cooler Temperature : 7 C
Measuring Line Purge Volume : 100 uL
Air Gap Volume : 0.1 uL
Loop Injection Type : Partial Loop
Rinse Method : Rinse port only
Rinse Time : 2 sec

<<Oven>>
Oven Model : CTO-20AC
Enable Oven : Use
Oven Temperature : 15 C
Maximum Temperature : 90 C
Ready Check : On

<<LC Time Program>>

Time	Module	Command	Value	Comment
3.00	Pumps	Pump B Conc.	8	
3.25	Pumps	Pump B Conc.	25	
17.50	Pumps	Pump B Conc.	25	
18.00	Pumps	Pump B Conc.	8	
20.00	Controller	Stop		

<<SFC A>>
SFC : SFC-30A
BPR Pressure : 100.0 bar
BPR Temperature : 50 C

<<SFC B>>
SFC : SFC-30A
BPR Pressure : 400.0 bar
BPR Temperature : 50 C

<<Sample Pretreatment>>
Mode : Standard

Sample Information

Data File : Amy-2_MeOH.lcd

Method

<<Pump>>

Mode : Binary gradient
Total Flow : 3.0000 mL/min
Pump C Flow : 0.1000 mL/min
B Conc. : 8.0 %
B Curve : 0
PressMax : 350 bar
PressMin : 0 bar
Pump C PressMax : 350 bar
Pump C PressMin : 0 bar
Solenoid Valve B Name : LPGE Unit
Solenoid Valve B : A
Compressibility Setting : Off
Pump A Shut Off Valve : Open

<<Autosampler>>

Autosampler Model : SIL-30AC
Enable Autosampler : Use
Rinse Type : External only
Sample Rack : Rack 1.5 mL 105 vials
Rinsing Volume : 500 uL
Needle Stroke : 52 mm
Control Vial Needle Stroke : 52 mm
Rinsing Speed : 35 uL/sec
Sampling Speed : 5.0 uL/sec
Sample Discharge Speed : 1.0 uL/sec
Measuring Line Purge Time : 10.0 min
Rinse Port R0 Purge Time : 10.0 min
Rinse Mode : Before and after aspiration
Rinse Dip Time : 0 sec
Cooler Temperature : 7 C
Measuring Line Purge Volume : 100 uL
Air Gap Volume : 0.1 uL
Loop Injection Type : Partial Loop
Rinse Method : Rinse port only
Rinse Time : 2 sec

<<Oven>>

Oven Model : CTO-20AC
Enable Oven : Use
Oven Temperature : 15 C
Maximum Temperature : 90 C
Ready Check : On

<<LC Time Program>>

Time	Module	Command	Value	Comment
3.00	Pumps	Pump B Conc.	8	
3.25	Pumps	Pump B Conc.	25	
17.50	Pumps	Pump B Conc.	25	
18.00	Pumps	Pump B Conc.	8	
20.00	Controller	Stop		

<<SFC A>>

SFC : SFC-30A
BPR Pressure : 100.0 bar
BPR Temperature : 50 C

<<SFC B>>

SFC : SFC-30A
BPR Pressure : 400.0 bar
BPR Temperature : 50 C

<<Sample Pretreatment>>

Mode : Standard

Sample Information

Data File	: Amy-3_IPA.lcd

Method

<<Pump>>

Mode	: Binary gradient
Total Flow	: 1.5000 mL/min
Pump C Flow	: 0.1000 mL/min
B Conc.	: 8.0 %
B Curve	: 0
PressMax	: 350 bar
PressMin	: 0 bar
Pump C PressMax	: 350 bar
Pump C PressMin	: 0 bar
Solenoid Valve B Name	: LPGE Unit
Solenoid Valve B	: A
Compressibility Setting	: Off
Pump A Shut Off Valve	: Open

<<Autosampler>>

Autosampler Model	: SIL-30AC
Enable Autosampler	: Use
Rinse Type	: External only
Sample Rack	: Rack 1.5 mL 105 vials
Rinsing Volume	: 500 uL
Needle Stroke	: 52 mm
Control Vial Needle Stroke	: 52 mm
Rinsing Speed	: 35 uL/sec
Sampling Speed	: 5.0 uL/sec
Sample Discharge Speed	: 1.0 uL/sec
Measuring Line Purge Time	: 10.0 min
Rinse Port R0 Purge Time	: 10.0 min
Rinse Mode	: Before and after aspiration
Rinse Dip Time	: 0 sec
Cooler Temperature	: 7 C
Measuring Line Purge Volume	: 100 uL
Air Gap Volume	: 0.1 uL
Loop Injection Type	: Partial Loop
Rinse Method	: Rinse port only
Rinse Time	: 2 sec

<<Oven>>

Oven Model	: CTO-20AC
Enable Oven	: Use
Oven Temperature	: 15 C
Maximum Temperature	: 90 C
Ready Check	: On

<<LC Time Program>>

Time	Module	Command	Value	Comment
3.00	Pumps	Pump B Conc.	8	
3.25	Pumps	Pump B Conc.	25	
17.50	Pumps	Pump B Conc.	25	
18.00	Pumps	Pump B Conc.	8	
20.00	Controller	Stop		

<<SFC A>>

SFC	: SFC-30A
BPR Pressure	: 100.0 bar
BPR Temperature	: 50 C

<<SFC B>>

SFC	: SFC-30A
BPR Pressure	: 400.0 bar
BPR Temperature	: 50 C

<<Sample Pretreatment>>

Mode	: Standard

Sample Information

Data File : Amy-3_MeOH.lcd

Method

<<Pump>>

Mode : Binary gradient
Total Flow : 1.5000 mL/min
Pump C Flow : 0.1000 mL/min
B Conc. : 8.0 %
B Curve : 0
PressMax : 350 bar
PressMin : 0 bar
Pump C PressMax : 350 bar
Pump C PressMin : 0 bar
Solenoid Valve B Name : LPGE Unit
Solenoid Valve B : A
Compressibility Setting : Off
Pump A Shut Off Valve : Open

<<Autosampler>>

Autosampler Model : SIL-30AC
Enable Autosampler : Use
Rinse Type : External only
Sample Rack : Rack 1.5 mL 105 vials
Rinsing Volume : 500 uL
Needle Stroke : 52 mm
Control Vial Needle Stroke : 52 mm
Rinsing Speed : 35 uL/sec
Sampling Speed : 5.0 uL/sec
Sample Discharge Speed : 1.0 uL/sec
Measuring Line Purge Time : 10.0 min
Rinse Port R0 Purge Time : 10.0 min
Rinse Mode : Before and after aspiration
Rinse Dip Time : 0 sec
Cooler Temperature : 7 C
Measuring Line Purge Volume : 100 uL
Air Gap Volume : 0.1 uL
Loop Injection Type : Partial Loop
Rinse Method : Rinse port only
Rinse Time : 2 sec

<<Oven>>

Oven Model : CTO-20AC
Enable Oven : Use
Oven Temperature : 15 C
Maximum Temperature : 90 C
Ready Check : On

<<LC Time Program>>

Time	Module	Command	Value	Comment
3.00	Pumps	Pump B Conc.	8	
3.25	Pumps	Pump B Conc.	25	
17.50	Pumps	Pump B Conc.	25	
18.00	Pumps	Pump B Conc.	8	
20.00	Controller	Stop		

<<SFC A>>

SFC : SFC-30A
BPR Pressure : 100.0 bar
BPR Temperature : 50 C

<<SFC B>>

SFC : SFC-30A
BPR Pressure : 400.0 bar
BPR Temperature : 50 C

<<Sample Pretreatment>>

Mode : Standard

Sample Information

Data File : Cellu-5_IPA.lcd

Method

<<Pump>>
Mode : Binary gradient
Total Flow : 1.5000 mL/min
Pump C Flow : 0.1000 mL/min
B Conc. : 8.0 %
B Curve : 0
PressMax : 350 bar
PressMin : 0 bar
Pump C PressMax : 350 bar
Pump C PressMin : 0 bar
Solenoid Valve B Name : LPGE Unit
Solenoid Valve B : A
Compressibility Setting : Off
Pump A Shut Off Valve : Open

<<Autosampler>>
Autosampler Model : SIL-30AC
Enable Autosampler : Use
Rinse Type : External only
Sample Rack : Rack 1.5 mL 105 vials
Rinsing Volume : 500 uL
Needle Stroke : 52 mm
Control Vial Needle Stroke : 52 mm
Rinsing Speed : 35 uL/sec
Sampling Speed : 5.0 uL/sec
Sample Discharge Speed : 1.0 uL/sec
Measuring Line Purge Time : 10.0 min
Rinse Port R0 Purge Time : 10.0 min
Rinse Mode : Before and after aspiration
Rinse Dip Time : 0 sec
Cooler Temperature : 7 C
Measuring Line Purge Volume : 100 uL
Air Gap Volume : 0.1 uL
Loop Injection Type : Partial Loop
Rinse Method : Rinse port only
Rinse Time : 2 sec

<<Oven>>
Oven Model : CTO-20AC
Enable Oven : Use
Oven Temperature : 15 C
Maximum Temperature : 90 C
Ready Check : On

<<LC Time Program>>

Time	Module	Command	Value	Comment
3.00	Pumps	Pump B Conc.	8	
3.25	Pumps	Pump B Conc.	25	
17.50	Pumps	Pump B Conc.	25	
18.00	Pumps	Pump B Conc.	8	
20.00	Controller	Stop		

<<SFC A>>
SFC : SFC-30A
BPR Pressure : 100.0 bar
BPR Temperature : 50 C

<<SFC B>>
SFC : SFC-30A
BPR Pressure : 400.0 bar
BPR Temperature : 50 C

<<Sample Pretreatment>>
Mode : Standard

Sample Information

Data File : Cellu-5_MeOH.lcd

Method

<<Pump>>
Mode : Binary gradient
Total Flow : 1.5000 mL/min
Pump C Flow : 0.1000 mL/min
B Conc. : 8.0 %
B Curve : 0
PressMax : 350 bar
PressMin : 0 bar
Pump C PressMax : 350 bar
Pump C PressMin : 0 bar
Solenoid Valve B Name : LPGE Unit
Solenoid Valve B : A
Compressibility Setting : Off
Pump A Shut Off Valve : Open

<<Autosampler>>
Autosampler Model : SIL-30AC
Enable Autosampler : Use
Rinse Type : External only
Sample Rack : Rack 1.5 mL 105 vials
Rinsing Volume : 500 uL
Needle Stroke : 52 mm
Control Vial Needle Stroke : 52 mm
Rinsing Speed : 35 uL/sec
Sampling Speed : 5.0 uL/sec
Sample Discharge Speed : 1.0 uL/sec
Measuring Line Purge Time : 10.0 min
Rinse Port R0 Purge Time : 10.0 min
Rinse Mode : Before and after aspiration
Rinse Dip Time : 0 sec
Cooler Temperature : 7 C
Measuring Line Purge Volume : 100 uL
Air Gap Volume : 0.1 uL
Loop Injection Type : Partial Loop
Rinse Method : Rinse port only
Rinse Time : 2 sec

<<Oven>>
Oven Model : CTO-20AC
Enable Oven : Use
Oven Temperature : 15 C
Maximum Temperature : 90 C
Ready Check : On

<<LC Time Program>>

Time	Module	Command	Value	Comment
3.00	Pumps	Pump B Conc.	8	
3.25	Pumps	Pump B Conc.	25	
17.50	Pumps	Pump B Conc.	25	
18.00	Pumps	Pump B Conc.	8	
20.00	Controller	Stop		

<<SFC A>>
SFC : SFC-30A
BPR Pressure : 100.0 bar
BPR Temperature : 50 C

<<SFC B>>
SFC : SFC-30A
BPR Pressure : 400.0 bar
BPR Temperature : 50 C

<<Sample Pretreatment>>
Mode : Standard
