# Supplementary material for: Subcritical Fluid Chromatography at Sub-Ambient Temperatures for the Chiral Resolution of Ketamine Metabolites with Rapid-Onset Antidepressant Effects
Source: Molecules. 2019 May 19;24(10):1927. doi: 10.3390/molecules24101927 (PMC6572699; doi:10.3390/molecules24101927)
Supplement: Supplementary file 1 [file molecules-24-01927-s001.zip › S3.pdf]

Sample Information

Data File : MM10µL_100$200bar_0%_8%_25%_001.lcd

Method

<<Pump>>

Mode	: Binary gradient
Total Flow	: 1.5000 mL/min
Pump C Flow	: 0.1000 mL/min
B Conc.	: 0.0 %
B Curve	: 0
PressMax	: 350 bar
PressMin	: 0 bar
Pump C PressMax	: 350 bar
Pump C PressMin	: 0 bar
Solenoid Valve B Name	: LPGE Unit
Solenoid Valve B	: C
Compressibility Setting	: Off

<<Autosampler>>

Autosampler Model	: SFE-30A
Heating Individually	: Off
Vessel 1 Temperature	: 35 C
Vessel 2 Temperature	: 35 C
Vessel 3 Temperature	: 35 C
Vessel 4 Temperature	: 35 C
Temperature Limit	: 93 C
Rinsing Needle After Extracting	: 2 sec

<<Oven>>

Oven Model	: CTO-20AC
Enable Oven	: Use
Oven Temperature	: 15 C
Maximum Temperature	: 90 C
Ready Check	: On

<<LC Time Program>>

Time	Module	Command	Value	Comment
3.00	SFE	Extraction Valve	Dynamic	
3.49	Supercritical Controller B		BPR Press.	400.0
3.49	Supercritical Controller A		BPR Press.	100.0
3.50	SFE	End		
3.51	Pumps	Pump B Conc.	0	
3.52	Pumps	Pump B Conc.	8	
6.50	Pumps	Pump B Conc.	8	
6.75	Pumps	Pump B Conc.	25	
12.50	Pumps	Pump B Conc.	25	
12.50	SFE	Wash		
12.51	Pumps	Pump B Conc.	100	
12.51	Pumps	Shut Off Valve(Pump A)	Close	
12.52	Pumps	Total Flow	1.5	
12.53	Pumps	Total Flow	0.4	
14.53	Pumps	Shut Off Valve(Pump A)	Open	
14.53	Pumps	Total Flow	0.4	
14.54	Pumps	Total Flow	0	
15.03	Pumps	Shut Off Valve(Pump A)	Open(Pump On)	
15.04	SFE	Extraction Valve	Static	
15.04	Pumps	Total Flow	0	
15.05	Pumps	Total Flow	1.5	
15.06	Pumps	Pump B Conc.	100	
15.07	Pumps	Pump B Conc.	3	
16.57	Controller	Stop		

<<SFC A>>

SFC	: SFC-30A
BPR Pressure	: 100.0 bar
BPR Temperature	: 50 C

<<SFC B>>

SFC	: SFC-30A
BPR Pressure	: 200.0 bar
BPR Temperature	: 50 C

Sample Information

Data File : MM10µL_100$200bar_0%_8%_25%_001.lcd

Method

<<Pump>>

Mode : Binary gradient
Total Flow : 1.5000 mL/min
Pump C Flow : 0.1000 mL/min
B Conc. : 0.0 %
B Curve : 0
PressMax : 350 bar
PressMin : 0 bar
Pump C PressMax : 350 bar
Pump C PressMin : 0 bar
Solenoid Valve B Name : LPGE Unit
Solenoid Valve B : C
Compressibility Setting : Off

<<Autosampler>>

Autosampler Model : SFE-30A
Heating Individually : Off
Vessel 1 Temperature : 35 C
Vessel 2 Temperature : 35 C
Vessel 3 Temperature : 35 C
Vessel 4 Temperature : 35 C
Temperature Limit : 93 C
Rinsing Needle After Extracting : 2 sec

<<Oven>>

Oven Model : CTO-20AC
Enable Oven : Use
Oven Temperature : 15 C
Maximum Temperature : 90 C
Ready Check : On

<<LC Time Program>>

Time	Module	Command	Value	Comment
3.00	SFE	Extraction Valve	Dynamic	
3.49	Supercritical Controller B		BPR Press.	400.0
3.49	Supercritical Controller A		BPR Press.	100.0
3.50	SFE	End		
3.51	Pumps	Pump B Conc.	0	
3.52	Pumps	Pump B Conc.	8	
6.50	Pumps	Pump B Conc.	8	
6.75	Pumps	Pump B Conc.	25	
12.50	Pumps	Pump B Conc.	25	
12.50	SFE	Wash		
12.51	Pumps	Pump B Conc.	100	
12.51	Pumps	Shut Off Valve(Pump A)	Close	
12.52	Pumps	Total Flow	1.5	
12.53	Pumps	Total Flow	0.4	
14.53	Pumps	Shut Off Valve(Pump A)	Open	
14.53	Pumps	Total Flow	0.4	
14.54	Pumps	Total Flow	0	
15.03	Pumps	Shut Off Valve(Pump A)	Open(Pump On)	
15.04	SFE	Extraction Valve	Static	
15.04	Pumps	Total Flow	0	
15.05	Pumps	Total Flow	1.5	
15.06	Pumps	Pump B Conc.	100	
15.07	Pumps	Pump B Conc.	3	
16.57	Controller	Stop		

<<SFC A>>

SFC : SFC-30A
BPR Pressure : 100.0 bar
BPR Temperature : 50 C

<<SFC B>>

SFC : SFC-30A
BPR Pressure : 200.0 bar
BPR Temperature : 50 C

Sample Information

Data File : MM10µL_100$200bar_0%_8%_25%_001.lcd

Method

<<Pump>>

Mode	: Binary gradient
Total Flow	: 1.5000 mL/min
Pump C Flow	: 0.1000 mL/min
B Conc.	: 0.0 %
B Curve	: 0
PressMax	: 350 bar
PressMin	: 0 bar
Pump C PressMax	: 350 bar
Pump C PressMin	: 0 bar
Solenoid Valve B Name	: LPGE Unit
Solenoid Valve B	: C
Compressibility Setting	: Off

<<Autosampler>>

Autosampler Model	: SFE-30A
Heating Individually	: Off
Vessel 1 Temperature	: 35 C
Vessel 2 Temperature	: 35 C
Vessel 3 Temperature	: 35 C
Vessel 4 Temperature	: 35 C
Temperature Limit	: 93 C
Rinsing Needle After Extracting	: 2 sec

<<Oven>>

Oven Model	: CTO-20AC
Enable Oven	: Use
Oven Temperature	: 15 C
Maximum Temperature	: 90 C
Ready Check	: On

<<LC Time Program>>

Time	Module	Command	Value	Comment
3.00	SFE	Extraction Valve	Dynamic	
3.49	Supercritical Controller B		BPR Press.	400.0
3.49	Supercritical Controller A		BPR Press.	100.0
3.50	SFE	End		
3.51	Pumps	Pump B Conc.	0	
3.52	Pumps	Pump B Conc.	8	
6.50	Pumps	Pump B Conc.	8	
6.75	Pumps	Pump B Conc.	25	
12.50	Pumps	Pump B Conc.	25	
12.50	SFE	Wash		
12.51	Pumps	Pump B Conc.	100	
12.51	Pumps	Shut Off Valve(Pump A)	Close	
12.52	Pumps	Total Flow	1.5	
12.53	Pumps	Total Flow	0.4	
14.53	Pumps	Shut Off Valve(Pump A)	Open	
14.53	Pumps	Total Flow	0.4	
14.54	Pumps	Total Flow	0	
15.03	Pumps	Shut Off Valve(Pump A)	Open(Pump On)	
15.04	SFE	Extraction Valve	Static	
15.04	Pumps	Total Flow	0	
15.05	Pumps	Total Flow	1.5	
15.06	Pumps	Pump B Conc.	100	
15.07	Pumps	Pump B Conc.	3	
16.57	Controller	Stop		

<<SFC A>>

SFC	: SFC-30A
BPR Pressure	: 100.0 bar
BPR Temperature	: 50 C

<<SFC B>>

SFC	: SFC-30A
BPR Pressure	: 200.0 bar
BPR Temperature	: 50 C

Sample Information

Data File : MM10µL_100$200bar_0%_8%_25%_001.lcd

Method

<<Pump>>

Mode : Binary gradient
Total Flow : 1.5000 mL/min
Pump C Flow : 0.1000 mL/min
B Conc. : 0.0 %
B Curve : 0
PressMax : 350 bar
PressMin : 0 bar
Pump C PressMax : 350 bar
Pump C PressMin : 0 bar
Solenoid Valve B Name : LPGE Unit
Solenoid Valve B : C
Compressibility Setting : Off

<<Autosampler>>

Autosampler Model : SFE-30A
Heating Individually : Off
Vessel 1 Temperature : 35 C
Vessel 2 Temperature : 35 C
Vessel 3 Temperature : 35 C
Vessel 4 Temperature : 35 C
Temperature Limit : 93 C
Rinsing Needle After Extracting : 2 sec

<<Oven>>

Oven Model : CTO-20AC
Enable Oven : Use
Oven Temperature : 15 C
Maximum Temperature : 90 C
Ready Check : On

<<LC Time Program>>

Time	Module	Command	Value	Comment
3.00	SFE	Extraction Valve	Dynamic	
3.49	Supercritical Controller B		BPR Press.	400.0
3.49	Supercritical Controller A		BPR Press.	100.0
3.50	SFE	End		
3.51	Pumps	Pump B Conc.	0	
3.52	Pumps	Pump B Conc.	8	
6.50	Pumps	Pump B Conc.	8	
6.75	Pumps	Pump B Conc.	25	
12.50	Pumps	Pump B Conc.	25	
12.50	SFE	Wash		
12.51	Pumps	Pump B Conc.	100	
12.51	Pumps	Shut Off Valve(Pump A)	Close	
12.52	Pumps	Total Flow	1.5	
12.53	Pumps	Total Flow	0.4	
14.53	Pumps	Shut Off Valve(Pump A)	Open	
14.53	Pumps	Total Flow	0.4	
14.54	Pumps	Total Flow	0	
15.03	Pumps	Shut Off Valve(Pump A)	Open(Pump On)	
15.04	SFE	Extraction Valve	Static	
15.04	Pumps	Total Flow	0	
15.05	Pumps	Total Flow	1.5	
15.06	Pumps	Pump B Conc.	100	
15.07	Pumps	Pump B Conc.	3	
16.57	Controller	Stop		

<<SFC A>>

SFC : SFC-30A
BPR Pressure : 100.0 bar
BPR Temperature : 50 C

<<SFC B>>

SFC : SFC-30A
BPR Pressure : 200.0 bar
BPR Temperature : 50 C

Sample Information

Data File : MM10μL_100$200bar_0%_8%_25%_001.lcd

Method

<<Pump>>

Mode	: Binary gradient
Total Flow	: 1.5000 mL/min
Pump C Flow	: 0.1000 mL/min
B Conc.	: 0.0 %
B Curve	: 0
PressMax	: 350 bar
PressMin	: 0 bar
Pump C PressMax	: 350 bar
Pump C PressMin	: 0 bar
Solenoid Valve B Name	: LPGE Unit
Solenoid Valve B	: C
Compressibility Setting	: Off

<<Autosampler>>

Autosampler Model	: SFE-30A
Heating Individually	: Off
Vessel 1 Temperature	: 35 C
Vessel 2 Temperature	: 35 C
Vessel 3 Temperature	: 35 C
Vessel 4 Temperature	: 35 C
Temperature Limit	: 93 C
Rinsing Needle After Extracting	: 2 sec

<<Oven>>

Oven Model	: CTO-20AC
Enable Oven	: Use
Oven Temperature	: 15 C
Maximum Temperature	: 90 C
Ready Check	: On

<<LC Time Program>>

Time	Module	Command	Value	Comment
3.00	SFE	Extraction Valve	Dynamic	
3.49	Supercritical Controller B		BPR Press.	400.0
3.49	Supercritical Controller A		BPR Press.	100.0
3.50	SFE	End		
3.51	Pumps	Pump B Conc.	0	
3.52	Pumps	Pump B Conc.	8	
6.50	Pumps	Pump B Conc.	8	
6.75	Pumps	Pump B Conc.	25	
12.50	Pumps	Pump B Conc.	25	
12.50	SFE	Wash		
12.51	Pumps	Pump B Conc.	100	
12.51	Pumps	Shut Off Valve(Pump A)	Close	
12.52	Pumps	Total Flow	1.5	
12.53	Pumps	Total Flow	0.4	
14.53	Pumps	Shut Off Valve(Pump A)	Open	
14.53	Pumps	Total Flow	0.4	
14.54	Pumps	Total Flow	0	
15.03	Pumps	Shut Off Valve(Pump A)	Open(Pump On)	
15.04	SFE	Extraction Valve	Static	
15.04	Pumps	Total Flow	0	
15.05	Pumps	Total Flow	1.5	
15.06	Pumps	Pump B Conc.	100	
15.07	Pumps	Pump B Conc.	3	
16.57	Controller	Stop		

<<SFC A>>

SFC	: SFC-30A
BPR Pressure	: 100.0 bar
BPR Temperature	: 50 C

<<SFC B>>

SFC	: SFC-30A
BPR Pressure	: 200.0 bar
BPR Temperature	: 50 C

Sample Information

Data File : MM10µL_100$200bar_0%_8%_25%_001.lcd

Method

<<Pump>>

Mode	: Binary gradient
Total Flow	: 1.5000 mL/min
Pump C Flow	: 0.1000 mL/min
B Conc.	: 0.0 %
B Curve	: 0
PressMax	: 350 bar
PressMin	: 0 bar
Pump C PressMax	: 350 bar
Pump C PressMin	: 0 bar
Solenoid Valve B Name	: LPGE Unit
Solenoid Valve B	: C
Compressibility Setting	: Off

<<Autosampler>>

Autosampler Model	: SFE-30A
Heating Individually	: Off
Vessel 1 Temperature	: 35 C
Vessel 2 Temperature	: 35 C
Vessel 3 Temperature	: 35 C
Vessel 4 Temperature	: 35 C
Temperature Limit	: 93 C
Rinsing Needle After Extracting	: 2 sec

<<Oven>>

Oven Model	: CTO-20AC
Enable Oven	: Use
Oven Temperature	: 15 C
Maximum Temperature	: 90 C
Ready Check	: On

<<LC Time Program>>

Time	Module	Command	Value	Comment
3.00	SFE	Extraction Valve	Dynamic	
3.49	Supercritical Controller B		BPR Press.	400.0
3.49	Supercritical Controller A		BPR Press.	100.0
3.50	SFE	End		
3.51	Pumps	Pump B Conc.	0	
3.52	Pumps	Pump B Conc.	8	
6.50	Pumps	Pump B Conc.	8	
6.75	Pumps	Pump B Conc.	25	
12.50	Pumps	Pump B Conc.	25	
12.50	SFE	Wash		
12.51	Pumps	Pump B Conc.	100	
12.51	Pumps	Shut Off Valve(Pump A)	Close	
12.52	Pumps	Total Flow	1.5	
12.53	Pumps	Total Flow	0.4	
14.53	Pumps	Shut Off Valve(Pump A)	Open	
14.53	Pumps	Total Flow	0.4	
14.54	Pumps	Total Flow	0	
15.03	Pumps	Shut Off Valve(Pump A)	Open(Pump On)	
15.04	SFE	Extraction Valve	Static	
15.04	Pumps	Total Flow	0	
15.05	Pumps	Total Flow	1.5	
15.06	Pumps	Pump B Conc.	100	
15.07	Pumps	Pump B Conc.	3	
16.57	Controller	Stop		

<<SFC A>>

SFC	: SFC-30A
BPR Pressure	: 100.0 bar
BPR Temperature	: 50 C

<<SFC B>>

SFC	: SFC-30A
BPR Pressure	: 200.0 bar
BPR Temperature	: 50 C
